# EFFECTS OF COGNITIVE DECLINE ON DIABETES SELF-MANAGEMENT EDUCATION AND CARE

**DOI:** 10.1093/geroni/igz038.139

**Published:** 2019-11-08

**Authors:** Shinduk Lee, Matthew L Smith, Marcia G Ory

**Affiliations:** 1 Center for Population Health and Aging, Texas A&M University, College Station, Texas, United States; 2 Texas A&M University, College Station, Texas, United States; 3 Texas A&M Center for Population Health and Aging, College Station, Texas, United States

## Abstract

People with diabetes experience a faster cognitive decline and have a greater risk for future dementia diagnoses. Cognitive impairment can negatively influence diabetes management activities. Diabetes self-management education (DSME) can enhance diabetes control, but limited evidence exists about the differential effects of DSME based on cognitive status. This study examines the moderation effects of cognition on the relationship between participation in DSME and diabetes management among older adults using Georgia 2017 BRFSS data (N=496). Primary outcomes were diabetes self-management (e.g., self-blood glucose monitoring, self-feet check, and physical activity) and clinical care (e.g., seeing a health professional for diabetes and A1C, feet, and eye exams). Multiple logistic regression models examined the effects of DSME and self-reported cognitive decline on diabetes care. Based on the Anderson-and-Newman Framework, all regression models were adjusted for predisposing (age, sex, race, ethnicity, and education), enabling (income, marital status, and health plan), and need (insulin treatment) factors. About 48% of participants participated in a DSME, and about 16% reported experiencing cognitive decline. DSME participation was positively associated with self-blood glucose monitoring (p=0.014), physical activity (p=0.024), seeing a health professional for diabetes (p=0.002), and feet exam (p=0.043), but cognitive decline was not significantly associated with most diabetes care (p>0.05). Further, no significant difference in DSME impact on diabetes care based on reported cognitive decline was observed (p>0.05). Findings suggest that DSME can benefit diabetes care among people with and without cognitive decline. Future research can expand upon impacts of rates and degrees of cognitive decline on program benefits.

